# Formulation, preparation of niosome loaded zinc oxide nanoparticles and biological activities

**DOI:** 10.1038/s41598-024-67509-5

**Published:** 2024-07-19

**Authors:** Hossein Rezaei, Alireza Iranbakhsh, Abbas Akhavan Sepahi, Amir Mirzaie, Kambiz Larijani

**Affiliations:** 1 Department of Biology, Science and Research Branch, Islamic Azad University, Tehran, Iran; 2 Department of Microbiology, Faculty of Biological Sciences, North Tehran Branch, Islamic Azad University, Tehran, Iran; 3 Department of Biology, Parand Branch, Islamic Azad University, Parand, Iran; 4 Department of Chemistry, Science and Research Branch, Islamic Azad University, Tehran, Iran

**Keywords:** Niosome, Zinc oxide nanoparticles, Niosome loaded Zn-NPs, Green synthesis method, Biological activities, Nanomedicine, Microbiology

## Abstract

In this study, zinc oxide nanoparticles (Zn-NPs) were prepared by the green synthesis method and loaded inside niosomes as a drug release system and their physicochemical and biological properties were determined. Zn-NPs were prepared by the eco-friendly green strategy, the structure, and morphological properties were studied and loaded into niosomes. Subsequently, different formulations of niosomes containing Zn-NPs were prepared and the optimal formulation was used for biological studies. Scanning electron microscope (SEM) and dynamic light scattering (DLS) were used to investigate the morphology and size of nanoparticles. Fourier transform infrared spectroscopy (FTIR) and UV–Vis were used to confirm the synthesis of Zn-NPs. Energy dispersive X-ray spectrometer (EDS) determined the elemental analysis of the Zn-NPs synthesis solution and the crystalline structure of Zn-NPs was analysed by XRD (X-Ray diffraction). Furthermore, Zn-NPs were loaded inside the niosomes, and their structural characteristics, entrapment efficiency (EE%), the release profile of Zn-NPs, and their stability also were assessed. Moreover, its antimicrobial properties against some microbial pathogens, its effect on the expression of biofilm genes, and its anticancer activity on the breast cancer cell lines were also determined. To study the cytocompatibility, exposure of niosomes against normal HEK-293 cells was carried out. In addition, the impact of niosomes on the expression of genes involved in the apoptosis (*Bcl2*, *Casp3*, *Casp9*, *Bax*) at the mRNA level was measured. Our findings revealed that the Zn-NPs have a round shape and an average size of 27.60 nm. Meanwhile, UV–Vis, FTIR, and XRD results confirmed the synthesis of Zn-NPs. Also, the EE% and the size of the optimized niosomal formulation were 31.26% and 256.6 ± 12 nm, respectively. The release profile showed that within 24 h, 26% of Zn-NPs were released from niosomes, while in the same period, 99% of free Zn-NPs were released, which indicates the slow release of Zn-NPs from niosomes. Antimicrobial effects exhibited that niosomes containing Zn-NPs had more significant antimicrobial and anti-biofilm effects than Zn-NPs alone, the antimicrobial and anti-biofilm effects increased 2 to 4 times. Cytotoxic effects indicated that when Zn-NPs are loaded into niosomes, the anticancer activity increases compared to Zn-NPs alone and has low cytotoxicity on cancer cells. Niosomes containing ZnNPs increased the apoptosis-related gene expression level and reduced the *Bcl2* genes. In general, the results show that niosomes can increase the biological effects of free Zn-NPs and therefore can be a suitable carrier for targeted delivery of Zn-NPs.

## Introduction

Recently, drug resistance in bacteria and cancer cells is one of the challenging issues and researchers are looking for a suitable strategy for treatment^1^. One of these solutions is the use of metal nanoparticles, especially zinc oxide nanoparticles (Zn-NPs)^2^. Zinc oxide is one of the zinc compounds that is recognized as safe by some organizations such as the World Health Organization (WHO)^3^. Zn-NPs are widely used in various industries such as food, health, environment, and medical industries. Moreover, Zn-NPs have antimicrobial applications against a wider range of bacteria and have received much attention in the last two decades^4^. The mechanism of antimicrobial action of Zn-NPs is similar to other metallic nanoparticles and acts mainly through the destruction of the bacterial cell wall^5^. The exact mechanism of Zn-NP action is not yet known, but there are many reports that these effects are caused by the high surface-to-volume ratio of nanoparticles, as well as entering the cell due to its small size, which destroys the membrane structure and DNA molecule^6^. In general, there are various chemical methods for the synthesis of Zn-NPs, however, the nanoparticles obtained from these methods due to the use of dangerous chemical substances, the methods have harmful effects on the environment and require high costs^7,8^. Therefore, it is necessary to find a suitable, inexpensive, and eco-friendly strategy without its residues being non-degradable, for the synthesis of Zn-NPs^9^. One of the methods that has recently attracted the attention of researchers is the biological method, and one of these biological methods is green synthesis, in which plant extract has the role of reduction and synthesis of nanoparticles^10^. One of the advantages of using plants is their harmlessness to the environment and their cheapness^11^. Also, because many plant species are natively available to researchers in many countries and can be grown in bulk without the need for specific nutrients, it is very easily available^12^. Another advantage of using plants is that the extract of plants has secondary compounds such as terpenes and terpenoids, which have reducing properties and accelerate the synthesis process of Zn-NPs^13^. Also, another challenge of using Zn-NPs is their targeted release, which can use nanocarriers such as niosomes^14^. Also, another pharmaceutical challenge is the targeted delivery of the drug to the cells, slow-release, reducing the side effects of the drugs, and increasing the stability and half-life of the drugs^15^.

Niosomes are formed as a new drug delivery system by self-assembly of non-ionic surfactants in aqueous medium^16^. Niosomes as drug carriers reduce toxicity and significantly increase therapeutic indicators. Niosomes can enclose the drug and reduce its toxicity in the body, enhance the stability of the drug, and use a lower dose of the drug^17^. Moreover, the advantages of using niosomes compared to other nanocarriers such as liposomes are the ability to load hydrophilic and hydrophobic drugs, lower price, and more stability^18^. So far, few studies have been conducted on the pharmaceutical use of metal nanoparticles in niosomes^19^. In one of the studies conducted by Federica Rinaldi et al., silver nanoparticles were trapped inside the niosome structure, their chemical and biological properties were investigated^20^. Their results demonstrated that the entrapment efficiency of AgNPs is around 1–4% and the rate of release of AgNPs from niosomes is slow. Another report is by Farideh Rezaie Amale, et al. who determined the properties of gold nanoparticles synthesized by the green synthesis method and made several formulations of niosomes containing gold nanoparticles. In this study, the entrapment efficiency was reported 34.49% ± 0.84, and the authors stated that with the increase in the concentration of niosomes, the cytotoxic effects also increased^21^.

One of the goals of this research is the preparation of Zn-NPs through the green synthesis route, the investigation of their physical and chemical properties, and finally, the loading of Zn-NPs into niosomes. The characteristics of the synthesized niosomes including size and morphology, their antimicrobial and anticancer effects were evaluated.

## Materials and methods

The surfactants (including Span 60, and Tween 60), 96% ethanol, DMSO, agar, Mueller Hinton broth, crystal violet solution, and chloroform were obtained from Merck. Cholesterol, penicillin, streptomycin, zinc nitrate, cell culture medium, Trypan blue, and MTT dye were prepared from Sigma Aldrich. Bacterial cells and cell lines were taken from the center of biological and genetic resources of Iran. RNA extraction and cDNA synthesis kits were purchased from Qiagen, United States.

### Plant collection and extraction

In this study, the aerial parts of *Artemisia scoparia* were obtained from the plant bank of Iran Biological Reserves Center with herbarium number 1326 and were approved by an expert botanist. First, plant parts should be dried in an environment away from light and powdered using a grinder. 10 g of herbal powder was mixed with 50 ml of distilled water and extracted by maceration method. The prepared extract was filtered by filter paper (Whatman, Germany).

### Synthesis of zinc oxide nanoparticles (Zn-NPs) by green method

We claim that all experiments (including plant collection) complied with relevant institutional, national, and international guidelines and legislation. To obtain Zn-NPs with high purity, 100 ml of zinc nitrate with a concentration of 1.5 mM was added to 20 ml of the aerial part extract of *A scoparia*, and then it was treated with 10 ml of sodium hydroxide (1 M). The resulting mixture was maintained at a temperature of 60 °C and on a stirrer (MS300HS, Jhal Tehiz, Iran). After 24 h, the formation of a white color was observed, which indicates the appearance of Zn-NPs. For further purification, the resulting solution was centrifuged at 13,000 rpm for 5 min (Sigma, model 2.16, USA), washed with distilled water, and then with ethanol and dried^22^.

### Preparation of niosome-loaded Zn-NPs

To prepare the niosome-loaded Zn-NPs using the thin layer hydration method, cholesterol and surfactants (Span 60 and Tween 60) with different Mol ratios were dissolved in 10 ml chloroform (Merck, Germany) were placed in a rotary evaporator (113, Evaplus, Italy) at 55 °C and 120 rpm for 20 min under vacuum conditions (Table 3). Then, in the second step, sterile distilled water and 1 mg/ml Zn-NPs were added to the balloon with a final volume of 5 ml, and the rotation was set at 120 rpm and 55 °C for 20 min without vacuum conditions. Finally, the formed lipid vesicles containing the ZnNPs were stored in glass vials at room temperature for 24 h. Then, to reduce the size of the manufactured niosomes, it was placed in a sonicator (Ultrasonic Homogenizer App, 100 H, Hilscher, Germany) with a power of 100 W for 4 periods of 15 s^23^. The final concentration of Zn-NPs in the formed niosome-loaded Zn-NPs was 1 mg/ml and based on different molar ratios of Span 60 and Tween 60, 6 formulations of niosomes were synthesized (Table 3).

### Evaluation of the entrapment efficiency (EE%)

The entrapment efficiency of the ZnNPs into niosomes was measured by centrifugation (using Wit Gamb laboratory technology device, Korea) at 13,000 rotations per minute (rpm) for 90 min. The free Zn-NPs remained in the supernatant and the trapped drug remained in the sediment. In the next step, to calculate the percentage of Zn-NPs entrapment in niosomes, the sediment obtained was dissolved with 5 ml of isopropyl alcohol to separate the niosome and release the entrapped drug. Then, 2 ml of the obtained solution was added to a volume of 25 ml with sterile distilled water, and its absorption rate as well as the absorption of the free drug in the supernatant with an ultraviolet–visible spectroscopic device (430 nm) (Lambda 25, Perkin) Elmer, USA) was read. Using the following formula, the entrapment efficiency percentage was calculated^24^:

$$ {\text{Entrapment}}\;{\text{percentage }} = {\text{ amount }}\;{\text{of }}\;{\text{free }}\;{\text{Zn - NPs }} - {\text{ amount }}\;{\text{of}}\;{\text{ primary}}\;{\text{ Zn - NPs }}/{\text{ amount }}\;{\text{of }}\;{\text{primary}}\;{\text{ Zn - NPs }} \times { 1}00 $$

### Characterization of nanoparticles

After 120 min of the reaction time of adding the *A. scoparia* extract to zinc nitrate and changing the color of the reaction, ultraviolet–visible spectroscopic analysis of Zn-NPs using a UV–Vis spectrometer (Agilent, Cary 300, Spectrophotometer, USA) between 200 and 700 nm was carried out. To check the appearance of ZnNPs and niosome-loaded Zn-NPs, a micrograph of nanoparticles was prepared by transmission electron microscope (TEM). To perform TEM, a drop of Zn-NPs was placed on a copper grid. After that, the samples were washed with distilled water and stained with 2% uranyl acetate. Finally, imaging of the samples was done at 80 kV. In addition, the size and morphological study of Zn-NPs synthesized by the green synthesis method and niosome-loaded Zn-NPs were carried out via scanning electron microscope (SEM). For SEM, nanoparticles were diluted with deionized water (1:100) and a drop of it was placed on a silicon wafer and dried for 24 h at a suitable temperature. After that, coated with a layer of 100 Å gold for 3 min under argon at a pressure of 0.2 atm and studied under a field emission SEM. XRD analysis of the Zn-NPs was done to determine the crystalline phases of the Zn-NPs as well as to measure their crystal constants. XRD allows us to know the type of crystallographic shape of Zn-NPs and an XRD test was carried out by an XRD machine (Explorer, GNR, Italy) with CuKa lamp radiation and scanned from 2° to 80°, 2θ. To determine the functional groups of molecular compounds and, as a result, determine the possible structure of the compounds, the FTIR test was considered. The FTIR spectra of Zn-NPs and niosome-loaded Zn-NPs were investigated in KBr discs using a PerkinElmer FTIR spectrophotometer (spectrum Two, USA). FTIR analysis was conducted in a scanning range of 4000 to 400 cm^−1^ in a fixed resolution of 4 cm^−1^ at room temperature. A solid sample was examined by the KBr method, and percent transmission was recorded.

To determine the composition of the elements in the sample and the purity of the product in certain parts of it and with the roles of distribution of the elements in the sample according to the imaging level, EDS analysis has been used. EDS analysis on single particles was carried out using Oxford Instrument, Incax-act, equipped with SEM (JEOL-FE SEM). The particle size, polydispersity index (PDI), and zeta potential were measured using a zeta sizer, DLS (Malvern Panalytical, Malvern, UK) at 25 °C. A tiny vessel was used for the testing of 1 ml of the specimen. Samples were diluted with a dilution factor of 1:1000 using double distillation water and size measurements were performed in triplicate and data are expressed as mean ± SD.

### In vitro drug release studies

The release studies of niosomes were conducted using a cellulose acetate dialysis bag (molecular weight cutoff 12 KDa). 2 ml of niosome-loaded ZnNPs and free ZnNPs solutions were placed in separate dialysis bags. Each dialysis bag was suspended in a graduated cylinder with 100 ml phosphate-buffered saline (PBS) and placed on stirrers. The vessel was placed over a magnetic stirrer (50 rpm) and the temperature was maintained at 37 °C ± 0.5 °C. 1 ml of the sample was used to determine Zn-NP concentration and replaced with the same amount of phosphate buffer. Their optical absorption was read at 340 nm and the drug release chart was drawn^25^.

### Stability of niosomes

One of the methods of checking the stability of niosomes is to monitor them during a certain time in terms of specific characteristics. Briefly, 1 ml of nisomes solution containing Zn-NPs was examined for size and EE% criteria for one month at two temperatures of 4 and 25 °C^26^.

### Antibacterial activity

#### Microdilution method

To investigate the antimicrobial effects, the broth micro-dilution test was used. The strains used were *Enterococcus fecalis* ATCC 29212, *Pseudomonas aeruginosa* ATCC 15442, *Escherichia coli* ATCC 25922, and *Staphylococcus aureus* ATCC 700698. These strains were obtained from the microbial bank of Pasteur Institute of Iran. For this purpose, different dilutions of free Zn-NPs and niosomes containing Zn-NPs were prepared (7.8–1000 µg/ml) and 100 µl of the dilutions were poured into the wells of a 96-well plate. Then, 5 µl of 0.5 McFarland's concentration of studied bacteria and 95 µl of broth medium were added to the wells. After overnight incubation at 37 °C, the absorbance of the wells was read at a wavelength of 600 nm. We consider the first well where no growth was observed as the MIC concentration. Also, the first concentration that the culture of that well in the solid culture medium is negative is as MBC concentration. In this test, culture medium without inoculation of microbial cells and well-containing culture medium and microbial cells were used as negative and positive controls, respectively. In addition, this test was repeated three times^27^.

#### Time kill assay

This test was conducted according to the method of Mansouri et al. (2021), in summary, a concentration of 10^6^ CFU/mL was prepared from the desired strains and added to the a 96-well plate and treated with a concentration of 1/2 MIC, optical density was read at a wavelength of 600 nm at different times^28^.

### Anti-biofilm activity

To quantitatively study the biofilm inhibitory effects of Zn-NPs encapsulated by niosome and free ZN-NPs, the microtitre plate method-based crystal violet assay was used. Then, a 24 h culture was prepared from each strain, and then inoculated into a Tryptic Soy Broth (TSB) medium containing 0.2 glucose, a microbial suspension was obtained, whose turbidity was consistent with 0.5 McFarland. Then, 100 µl of each bacterial suspension were added to each of the wells of the plate, followed by 50 µl of niosome-loaded Zn-NPs and free Zn-NPs at sub-MIC concentrations, and then the plate was incubated for 24 h at 37 °C. After 24 h, the solution on the wells was removed and each well was washed 3 times with sterile physiological serum. Then, the bacteria attached to the wall were fixed with 250 µl of 96% ethanol. After 15 min, the contents of the well were emptied. After drying the plates, 200 µl of crystal violet dye were added (for 15 min). After this time, the extra dyes were washed using sterile distilled water. After drying the plates, biofilm was quantitatively measured by adding 200 µl of 33% acetic acid to each well, and its absorbance at 570 nm wavelength was read by ELISA Reader (JASCO, V-530, Japan)^28^.

### Biofilm gene expression analysis

To measure the expression of biofilm genes that were exposed to nanoparticles, first, the microbial cells were treated with ½ MIC concentration of nanoparticles and the total RNA of the cells was extracted using the high pure kit (Roche, Germany) according to the relevant instructions. The quality and quantity of RNA was determined by spectrophotometry of Nanodrop. Due to the instability of the extracted RNA, the extracted RNA was quickly converted to cDNA by the cDNA synthesis kit (Fermentase, Lithuania). After that, the Real-Time PCR method was used to evaluate the expression of *ndvB*, *fimH*, *icaD*, and *agg* biofilm genes. During PCR, by increasing the number of DNA copies, the light emitted from the fluorescent markers increases, which can be measured, and since the expression of the *16S rRNA* gene in the cell is stable, we use this gene as a housekeeping gene. The sequence of the primers used is listed in Table 1^29^:

**Table 1:** Primer sequence of genes involved in biofilm formation used in this research.

Gene	Primer sequence (5′ to 3′)	Ref.
*ndvB*	F 5′ GGCCTGAACATCTTCTTCACC-3′ R 5′ GATCTTGCCGACCTTGAAGAC-3′	^30^
*fimH*	F 5′ GCTGTGATGTTTCTGCTCGT-3′ R AAAACGAGGCGGTATTGGTG-3′	^30^
*icaD*	F ATGGTCAAGCCCAGACAGAG-3′ R AGTATTTTCAATGTTTAAAGCAA-3′	^30^
*Agg*	F CACGTAATTCTTGCCCACCA-3′ R AAACGGCAAGACAAGTAAATA-3′	30

### Cell culture

In this study, breast cancer cell lines including MDA-MB 361, MCF-7 and T47D cells and normal HEK-293 cells were purchased from the Pasteur Institute cell bank of Iran. These cell lines were cultured in a CO_2_ incubator at a temperature of 37 °C with 5% CO_2_ (passage number 1). Also, the contamination of the cells was checked in terms of mycoplasma and they were negative. To culture cells, from fresh medium (RPMI1640) containing 10% FBS and 1% antibiotic (penicillin/streptomycin) was used. When the cells reached 90–95% confluence, the medium was aspirated and the cell monolayer was washed three times with sterile phosphate-buffered saline. Cell monolayers were treated with 1 mL of 0.25% (w/v) trypsin–EDTA, briefly incubated at 37 °C, and observed microscopically to ensure complete cell detachment.

### Cytotoxicity assay and biocompatibility

The MTT method was used to determine the growth-inhibitory effects of niosomes on cancer cells. Briefly, after filling the flask containing the breast cancer cell lines including MDA-MB 361, MCF-7, and T47D cells, the cells were trypsinized and 5000 cells were poured into the wells of the cell culture flask (100 μl per well) and placed in a CO_2_ incubator. After removing the supernatant solution, the wells were exposed to various concentrations of niosomes (31.25, 62.5, 125, 250, 500, and 1000 μg/ml) and incubated again for 24 h. After that, they were treated with MTT dye and incubated in the dark for 4 h. Finally, the OD of the wells was read using an ELISA reader at 540 nm wavelength, and the survival rate of the cells was calculated using the following formula^30^:

Percentage of cell survival = optical density of control cells/optical density of treated cells. Also, to check the cytocompatibility of the synthesized niosomes, its cytotoxicity was investigated on the normal HEK-293 cell line.

### Measurement of apoptosis gene expression

Investigating the expression of some genes related to apoptosis at the mRNA level is one of the ways to study apoptosis induction. In this study, breast cancer cells were treated with niosomes containing Zn-NPs and Zn-NPs alone, and the expression levels of apoptotic genes *Bax*, *Bcl2*, *Casp3*, and *Casp9* were investigated using the Real-Time PCR method. In this study, the *GAPDH* was considered as a housekeeping gene, and the primers of the studied genes are given in Table 2^27^:

**Table 2:** The primer sequence of the target genes that were used to evaluate the expression of apoptotic genes.

Gene	Primer sequence	Ref.
*Bax*	5′-TTGCTTCAGGGTTTCATCCAG-3′ 5′-AGCTTCTTGGTGGACGCATC-3′	^30^
*Casp3*	5′-CATACTCCACAGCACCTGGTTA-3, 5′-ACTCAAATTCTGTTGCCACCTT-3′	^30^
*Casp9*	5′-CATATGATCGAGGACATCCAG-3 5′-TTAGTTCGCAGAAACGAAGC-3′	^30^
*Bcl2*	5′TGTGGATGACTGAGTACCTGAACC3′ 5′-CAGCCAGGAGAAATCAAACAGAG	^30^
*GAPDH*	5′-CGTCTGCCCTATCAACTTTCG-3′ 5′-CGTTTCTCAGGCTCCCCTCT-3′	^30^

### Statistical analysis

The data obtained from this study were put into GraphPad Prism software and the One-Way ANOVA test was used according to the normality of the data distribution, the comparison of the treatments and the control group was analyzed and the P-value was calculated. The significance level was considered at *p* < 0.05.

### Ethics approval

The authors of this article state that all methods are reported in accordance with ARRIVE guidelines (https://arriveguidelines.org). All protocols were performed by the Ethical Committee and Research Deputy of the Islamic Azad University, Science and Research Branch, Iran (IR.IAU.REC.1401.128).

## Results and discussion

### Preparation of Zn-NPs and niosomes loaded ZnNPs

**Figure 1:**
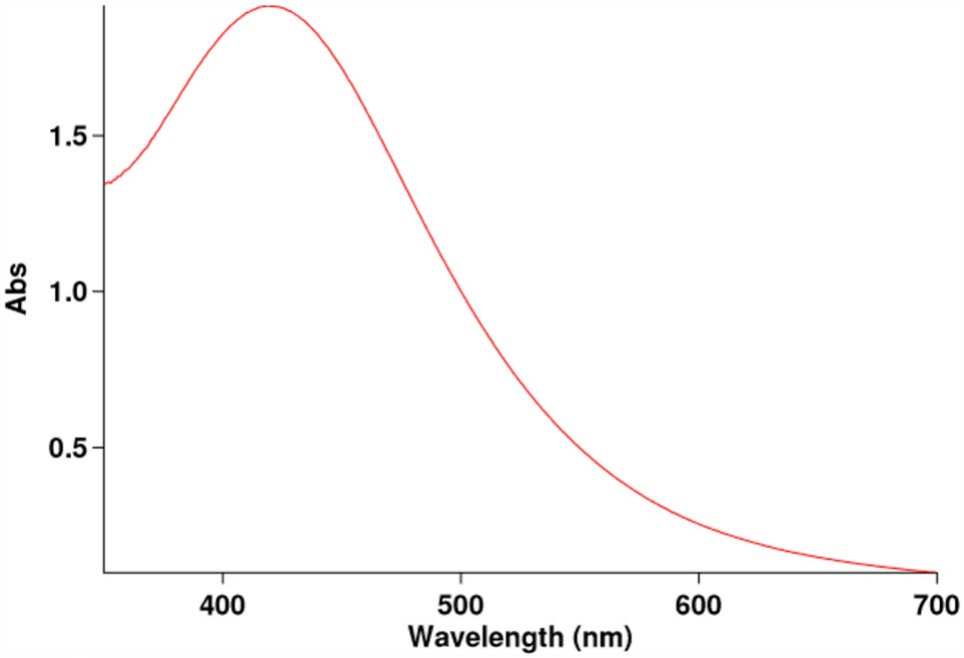
UV–Vis analysis of green synthesized Zn-NPs. The existence of a peak at the wavelength of 430 nm for Zn-NPs was confirmed by a UV–vis spectrometer.

During the Zn-NPs phytosynthesis process, the *A. scoparia* extract is mixed with zinc nitrate salt and causes its reduction. One of the characteristics of the reduction of zinc nitrate salt and the synthesis of Zn-NPs is the color change of the reaction and spectrometry^22^. The existence of a peak at the wavelength of 430 nm for Zn-NPs was confirmed by a UV–vis spectrometer (Fig. 1) (The concentration of Zn-NPs used for the UV test was 1 mg/ml). In this study, to achieve the optimal formulation of niosomes, based on variables such as the molar ratio of Tween 60, Span 60, and cholesterol, the synthesis of niosomes was formulated and 6 formulas of niosomes were formed with a sonication time of 6 min (Tables 3, 4). According to the results obtained in different niosomal formulations, the F2 formulation was considered the optimal formulation in terms of size and EE%, and the rest of the study was carried out with this formulation.

**Table 3:** Formulations of niosome loaded Zn-NPs.

Formulation	Zn-NPs (mg/ml)	Mol concentration of lipid	Surfactant	Mol ratio of Span60: Tween60	Mol ratio of surfactant to cholesterol	Sonication duration (min)
F1	1	200 µmol	Span 60	100:0	1:1	6
F2	1	200 µmol	Span 60	50:50	1:1	6
F3	1	200 µmol	Span 60	0:100	1:1	6
F4	1	200 µmol	Span 60	100:0	2:1	6
F5	1	200 µmol	Span 60	0:100	2:1	6
F6	1	200 µmol	Span 60	50:50	2:1	6

**Table 4:** The physico-chemical characteristics of niosome-loaded Zn-NPs (mean ± SD, n = 3).

Formulation	EE (%)	Size (nm)	PDI	Zeta potential (mV)
F1	24.65 ± 1.4	335.9 ± 6.59	0.299 ± 0.004	− 26.34 ± 2.38
F2	31.26 ± 1.89	256.6 ± 12	0.136 ± 0.006	− 23.77 ± 0.64
F3	18.33 ± 2.34	315 ± 4.89	0.238 ± 0.007	− 16.26 ± 4.46
F4	15.56 ± 1.94	429 ± 13.23	0.313 ± 0.003	− 25.15 ± 3.53
F5	22.34 ± 2.49	465 ± 5.69	0.173 ± 0.004	− 43.58 ± 5.41
F6	19.68 ± 1.13	389 ± 3.77	0.338 ± 0.006	− 16.36 ± 1.23

### Characterization of Zn-NPs and niosomes loaded Zn-NPs

**Figure 2:**
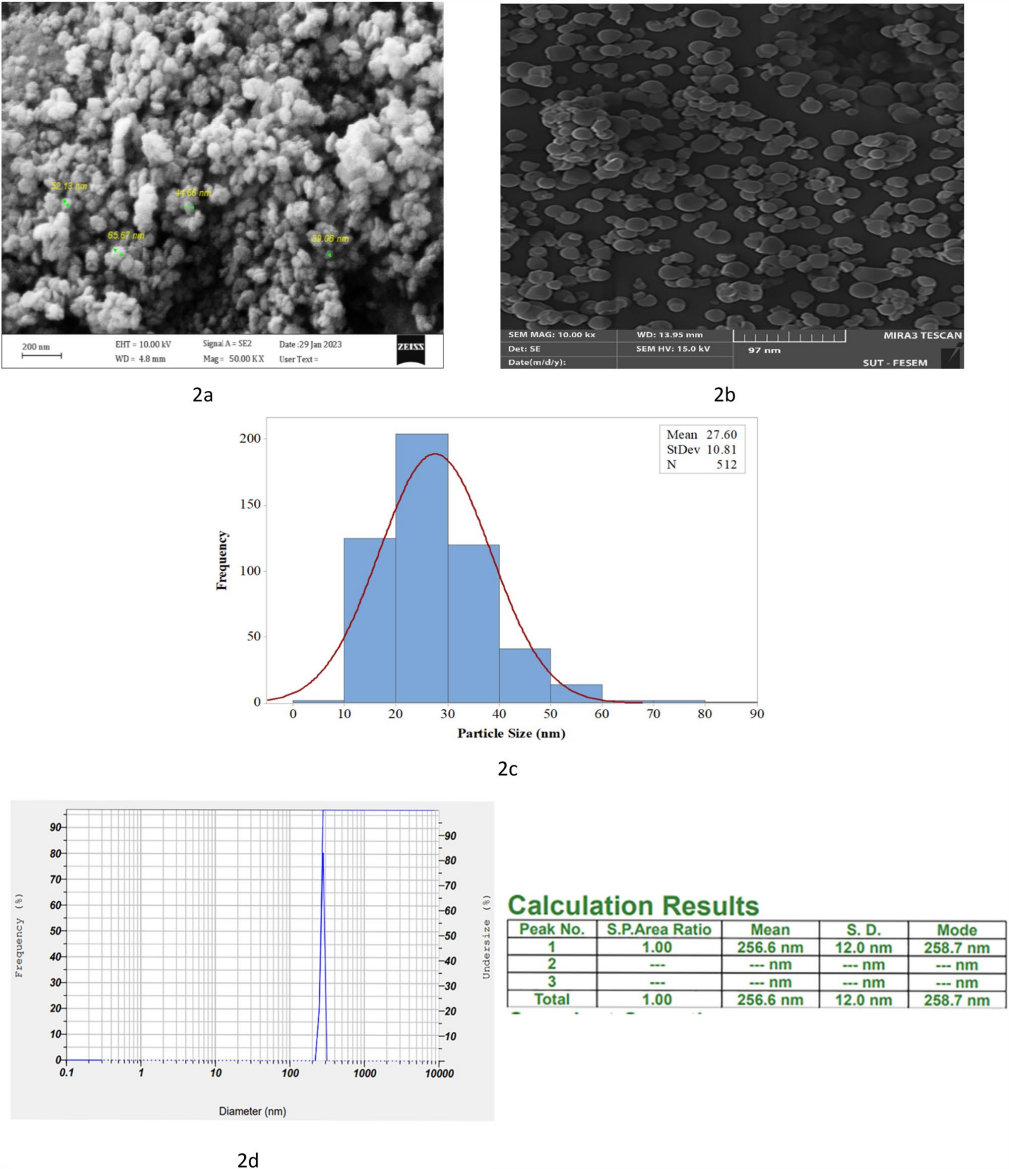
SEM micrograph of Zn-NPs (**a**), SEM analysis of niosome loaded Zn-NPs (**b**), Size distribution of synthesized Zn-NPs (**c**), DLS analysis of niosome loaded Zn-NPs (**d**).

The size and morphology of Zn-NPs and niosomes containing Zn-NPs were evaluated through SEM and DLS. Our findings indicated that Zn-NPs and niosomes loaded Zn-NPs had a spherical structure (Fig. 2a,b). Also, the average size of Zn-NPs was found to be 27.60 nm (Fig. 2c) and niosomes containing Zn-NPs were 256.6 ± 12 nm (Fig. 2d). Moreover, the zeta potential of optimal formulation of niosomes (F2) was -23.77 ± 0.64, showing a high formulation stability.

**Figure 3:**
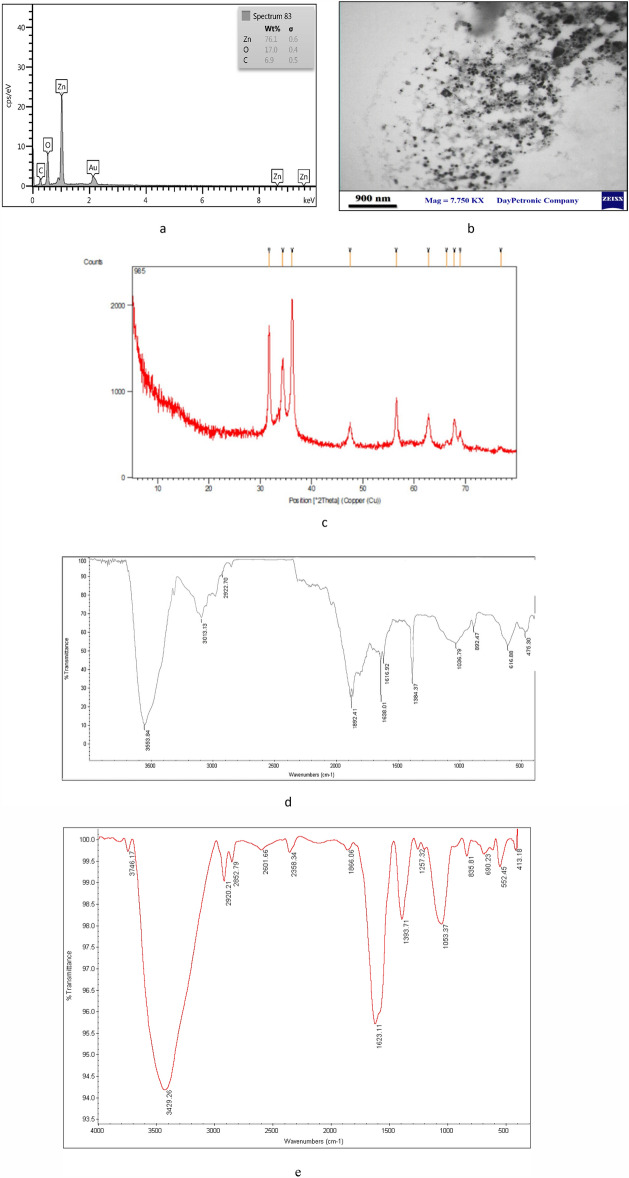
EDS analysis of Zn-NPs (**a**), TEM analysis of Zn-NPs (**b**), XRD analysis of ZN-NPs (**c**), FTIR analysis of niosome loaded Zn-NPs (**d**), FTIR analysis of Zn-NPs (**e**).

In some places micrographs of SEM, nanoparticles can be seen in clumps, which can be removed by sonication. Also, TEM results confirmed the spherical structure of Zn-NPs (Fig. 3b). The EDS method was used for the elemental analysis of the synthesized Zn-NPs, and the results show that 76.1% of the reaction mixture contains zinc (Fig. 3a). XRD analysis was performed to prove the crystalline structure of Zn-NPs. The spectrum pattern obtained from the X-ray at θ angle had peaks of 46.56, 36.29, 34.45, 33.28, and 33.8, which is consistent with the X-ray diffraction pattern of ZnO (Fig. 3c). The additional peaks observed in XRD may be due to the plant extract that was used in the synthesis of nanoparticles. In the FTIR results, peaks are observed that are related to the constituent compounds of niosomes. The peak at 475–616 cm^−1^ corresponds to ZN-NPs formation and is attributed to the metal–oxygen component. The peaks of 3555 and 3013 cm^−1^ are related to the hydroxyl groups in the plant extract, which are possibly related to the phenolic and alcoholic compounds of the extract. The 2922 cm^−1^ peak corresponds to the C–H stretching, which can be seen in the Span 60 structure (Fig. 3e,d).

### Drug release test

**Figure 4:**
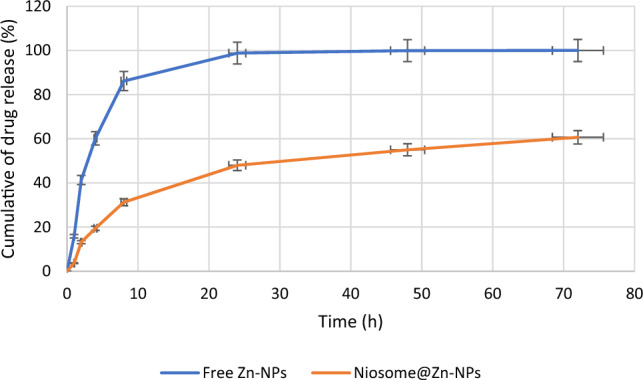
Release of Zn-NPs from niosomes compared with free form. This test was repeated 3 times and as can be seen, Zn-NPs loaded in niosomes have a slower release pattern compared to free Zn-NPs.

The dialysis bag method was used for the study of the release pattern of Zn-NPs from niosomes, and the release medium was PBS medium, and this test was completed within 72 h. In this study, the dialysis bag was placed in the PBS solution to take samples at different times and check the release pattern of Zn-NPs. The results showed that the rate of release of Zn-NPs in niosomal form was lower than that of Zn-NPs in the free form during 72 h. In addition, 86.15% of free Zn-NPs were released in the medium during the first 8 h, but for niosome containing Zn-NPs, 31.26% of the Zn-NPs were released within 8 h (Fig. 4). As can be seen in Fig. 2, the release of Zn-NPs from niosomes is done in 2 steps: as the results show, in the early hours, the release of Zn-NPs is continuous and happens more than in other hours. Gradually, in other hours until the end (72 h), the release becomes slower and the amount of release decreases. In general, there is no direct relationship between EE% and drug release. It means that the lower the EE%, the faster the release^31^. In this study, because the size of Zn-NPs (89.6 ± 3.8 nm) is small, the loading rate is reduced (which was equal to 31.26%), which leads to the rapid release of Zn-NPs. Studies show that the rate of drug release enclosed in niosomes also depends on the niosome composition^32^. The more rigid the niosomes are, the slower the release, and there are many reports that the niosomes prepared using Span 60, Tween 60, and cholesterol have a tighter structure and the drug release occurs slowly^33^. One of the similar researches has been done by Rinaldi et al., who trapped silver nanoparticles inside niosomes and studied their structural characteristics. The findings of this study showed the low EE% of silver nanoparticles loading and their release becomes much slower. Also, niosomes made of Span 20 and Tween 20 were stable in water, bovine serum, and human serum mediums^34^. The low percentage of loading has also been reported by other researchers, Rezaie Amale et al. reported that the loading rate of gold nanoparticles inside niosomes was equal to 34.49 ± 0.84% and the in vitro drug release test showed that after 8 h, 59 ± 2.0% of gold nanoparticles were released from niosomes. However, the free gold nanoparticles were released by 95 ± 1.0% during this time, which shows that niosomes can slow down the release^21^.

### Stability test

**Figure 5:**
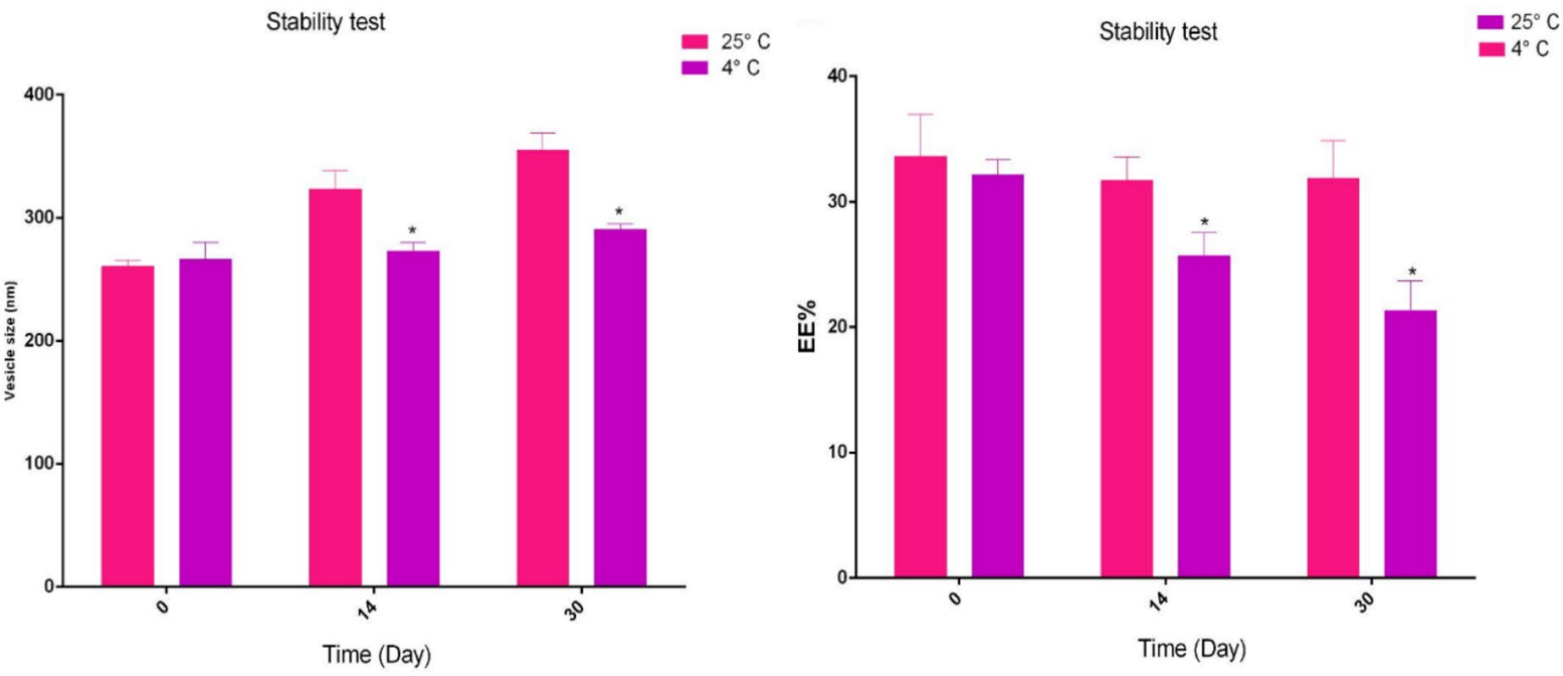
Stability of synthesized niosome samples at 4 °C and 25 °C based on EE% and vesicle size criteria. The test is repeated 3 times. *p < 0.05.

One of the characteristics of niosomes is their stability over time, and for this reason, the stability test for niosomes was carried out for 30 days at two temperatures of 4 °C and 25 °C, and the two characteristics of size and EE% were considered. The results of our study showed that the niosomes stored at the temperature of the refrigerator 4 °C, had more stability than the niosomes that were stored at the temperature of 25 °C, that is, their size and EE% had low changes. The noteworthy point is that over time, the instability of niosomes was obtained due to the increase in size and decrease in EE%. One of the mechanisms of increasing the size of niosomes with increasing time is the fusion of niosomes together. Also, studies show that times for hydration, sonication and introducing cholesterol into the niosome structure have a significant effect on its stability. In general, according to the results shown in Fig. 5, it can be concluded that niosomes are more stable at 4 °C fewer structural changes were observed and it is suggested to use this temperature to store niosomes. However, niosomes are more stable compared to other drug delivery systems such as liposomes, but as mentioned, the type of synthesis method, the type of surfactant, and the cholesterol content are effective in their stability.

### Antibacterial activity

#### Microdilution test

In this study, the antimicrobial effects of Zn-NPs containing niosomes, free Zn-NPs, and free niosomes were investigated against the microbial strains including *S. aureus, E. faecalis, E. coli*, and *P. aeruginosa*. The results revealed that free Zn-NPs had an MIC between 31.25 and 62.5 µg/ml, However, niosomes loaded Zn-NPs had an MIC between 7.8 and 15.62 µg/ml, which indicated an increase in antimicrobial effects by at least 2 times. Also, the MBC value of niosomes was much lower than the MBC of free Zn-NPs. The results showed that free niosomes did not have significant antimicrobial effects against pathogenic bacteria (Table 5). Research studies have shown that the mechanism of Zn-NPs antimicrobial effects is due to the creation of pores on the cell membrane of microbial cells, or some researchers have indicated that Zn-NPs can create toxic oxygen radicals (ROS) inside microbial cells^35^. One of the other mechanisms of the antimicrobial effects of Zn-NPs is to destroy the balance of the entry and exit of minerals due to the entry of Zn-NPs across the cell membrane, as well as the leakage of intracellular proteins and enzymes, inhibition of cell growth and death^36^. It is noteworthy that when Zn-NPs are loaded into niosomes, the antimicrobial effects increase. Numerous reports show that one of the reasons for decreased MIC value for niosomes and therefore the enhancement of bactericidal activity is due to the interaction of the bacterial membrane with the lipid structure of niosomal vesicles and the targeted delivery of Zn-NPs into the bacterial cell. Bacterial cell membrane interaction with niosomes takes place through three mechanisms: membrane integration, contact diffusion, and adsorption^28^. Iqbal et al., synthesized Zn-NPs by plant extract and investigated their antimicrobial effects on some microbial pathogens. The findings of this research showed that the MIC of Zn-NPs was 75 µg/ml for *P. aeruginosa* and 37.5 µg/ml for other bacteria studied^37^. Umavathi et al., synthesized Zn-NPs using the green synthesis method by aqueous extract of *Parthenium hysterophorus* and evaluated its activity on pathogenic bacteria and fungi. The researchers observed that Zn-NPs had significant antimicrobial effects against *E. coli* and stated that the antimicrobial potential of Zn-NPs is due to the attachment of the Zn-NPs to the cell membrane which has a negative charge and is attached to sulfur-containing amino acids and can disrupt the electron transport chain and DNA replication^38^.

**Table 5:** The MIC and MBC values of niosome loaded Zn-NPs and free Zn-NPs.

Bacteria	MIC of niosome loaded ZN-NPs/free Zn-NPs (µg/ml)	Sub-MIC of niosome loaded ZN-NPs/free Zn-NPs (µg/ml)	MBC of niosome loaded ZN-NPs/free Zn-NPs (µg/ml)
*P. aeroginosa*	15.62/62.5	7.8/31.25	31.25/125
*E. coli*	15.62/31.25	7.8/15.62	15.62/62.5
*S. aureus*	15.62/62.5	7.8/31.25	31.25/125
*E. fecalis*	7.8/31.25	< 7.8/15.62	7.8/62.5

#### Time kill assay

**Figure 6:**
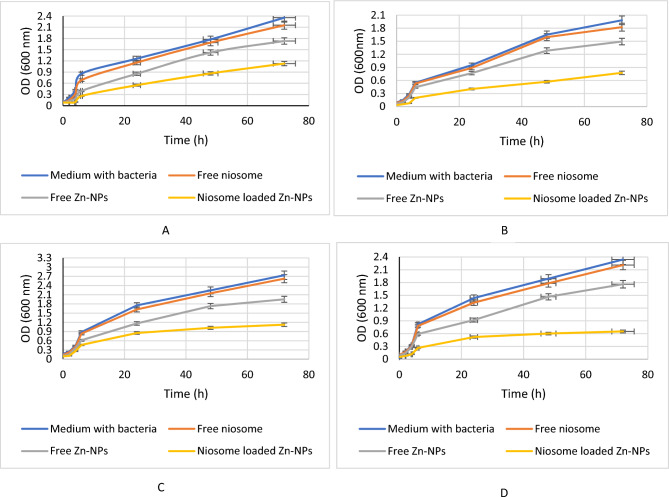
The antimicrobial effects of niosomes containing Zn-NPs and free Zn-NPs and free niosomes by time-kill assay method in a period of 72 h against *E. coli* (**A**), *P. aeruginosa* (**B**), *S. aureus* (**C**), *E. fecalis* (**D**). The results showed that niosomes containing Zn-NPs had more growth inhibitory effects. The tests were repeated triplicate and the mean ± SD was used.

To further study the kinetics of the antibacterial potential of niosomes loaded Zn-NPs and Zn-NPs alone, the time kill assay was considered. As you can see in Fig. 6, as a result of the treatment of bacteria with niosomes, the optical density is lower than other treatments in a period of 72 h and can inhibit the growth of microbial pathogens.

### Anti-biofilm activity

**Figure 7:**
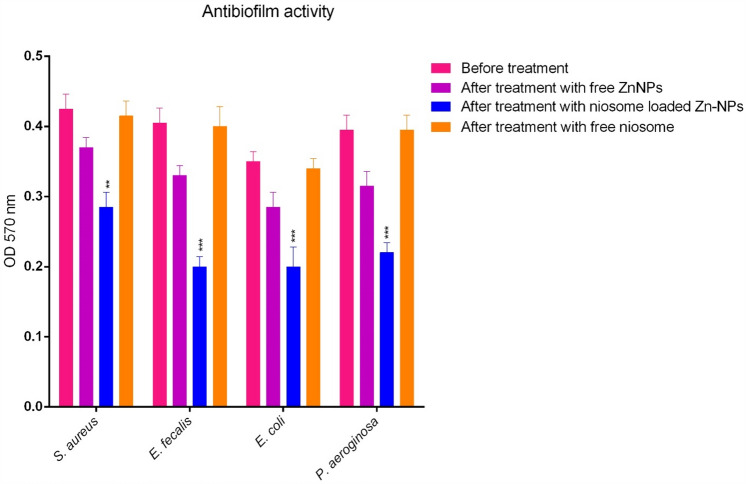
Biofilm inhibitory potential of niosome loaded Zn-NPs, Zn-NPs alone and empty niosomes. As the results show, niosomes reduce biofilm formation in pathogenic microbial strains (*p* < 0.001***, *p* < 0.01**, n = 3)**.**

In this test, the biofilm inhibition effects of niosome's optimal formulation were compared with Zn-NPs alone. Biofilm formation is one of the pathogenic mechanisms of microbial pathogens and in this test, which used the crystal violet colorimetric method, bacterial cells were exposed to ½ MIC values. We observed that niosomes can destroy the biofilm formed by microbial cells significantly (2–4 times) compared to free Zn-NPs (Fig. 7). The anti-biofilm effects of niosomes have been reported in many studies. The effects of biofilm inhibition by streptomycin-containing niosomes were investigated by Mansouri et al. who observed that niosomes can have two to fourfold anti-biofilm effects and even at concentrations 4 to 8 times lower than the MIC of niosomes, can eradicate biofilms. Some studies have shown that one of the mechanisms of the anti-biofilm effects of niosomes is the electrostatic binding of niosomes that have a positive charge to the surface of biofilms that have a negative charge and targeted drug release into the biofilm structure^39^.

### Impact of niosome on biofilm gene expression

**Figure 8:**
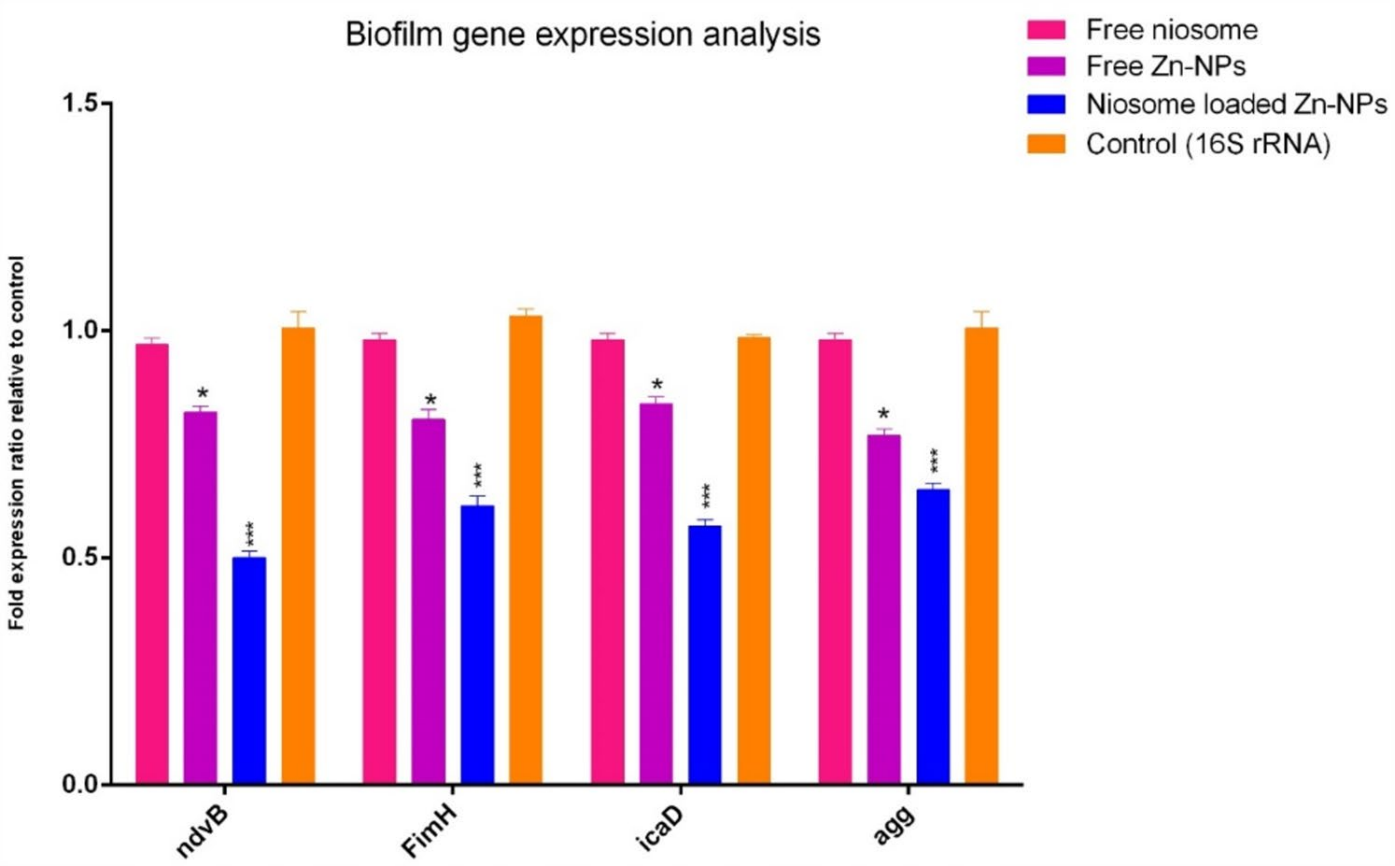
Biofilm gene expression analysis in pathogenic strains after treatment with niosome-loaded Zn-NPs, free Zn-NPs, and free niosome. The gene used as a control in this test was *16S rRNA*. The results are repeated triplicate. (*p* < 0.001***, *p* < 0.05*, n = 3)**.**

The expression of biofilm genes in bacteria plays an important role in the formation of biofilm, so to investigate the impact of niosomes on the expression of biofilm genes, methods based on gene expression at the mRNA level such as RT-PCR can be used. In this test, the strains were exposed to ½ MIC value of nanoparticles, and the expression level of biofilm-related genes was studied. Our findings demonstrated that a significant decrease in the expression of biofilm genes by niosomes has occurred, and this can confirm the reduction of the ability of biofilm formation in the studied strains (Fig. 8). Our findings are consistent with many studies in that niosomes inhibit biofilm formation.

Khaleghian et al. investigated the biofilm inhibitory effect niosomes encapsulated curcumin and the effects on the expression of genes involved in biofilm formation. Another finding of the study is a significant reduction in biofilm formation potential in a targeted manner and significantly reduced the expression of biofilm-forming genes in *S. aureus* strains^29^. Studies have shown that some metal nanoparticles can increase and inhibit the transcription of cell factors, and through blocking the transcription of biofilm-forming genes, the expression of genes decreases. Shakerimoghaddam et al. investigated the anti-biofilm effects of Zn-NPs on uropathogenic *E. coli* biofilm-forming bacteria. The point that the researchers of this research stated was that Zn-NPs can reduce biofilm formation and reduce the flu gene expression, which is effective in *E. coli* for initiation of attachment to surface and biofilm formation^40^.

### Cytotoxicity evaluation and cytocompatibility

**Figure 9:**
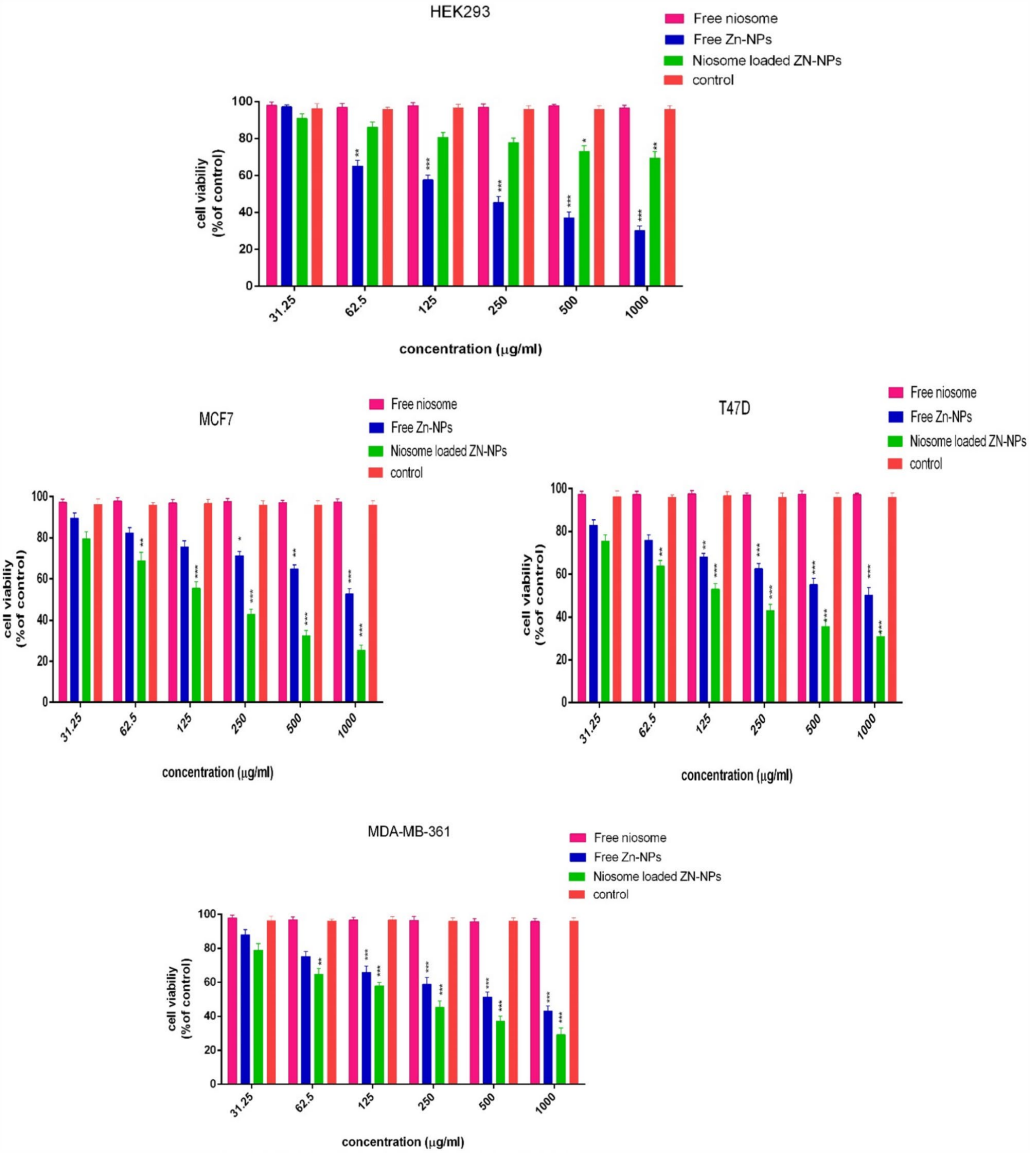
Cell viability measurement of breast cancer cell lines after treatment for 48 h using MTT test. HEK293 cell line was utilized for the cytocompatibility test (*p* < 0.001***, *p* < 0.01**, *p* < 0.05*, n = 3)**.**

In this study, the cytotoxic effects of niosome-loaded Zn-NPs and free Zn-NPs against MDA-MB 361, MCF-7, and T47D cancer cells were investigated. The findings showed that niosomes containing Zn-NPs could inhibit cell growth more than Zn-NPs alone and the increase in cytotoxic effects was dose-dependent and the cytotoxic effects increased with increasing concentration. The greatest cytotoxicity was related to the concentration of 1000 µg/ml and at this point, the cell survival rate was equal to 25.19 ± 1.21, 31.24 ± 1.67 and 29.64 ± 2.31 in T47D, MCF-7, and MD-MBA 361 cell lines, respectively. In the case of cells treated with free Zn-NPs, the cell survival rate was equal to 61.13 ± 1.33, 49.61 ± 1.59 and 43.13 ± 2.31 in T47D, MCF-7, and MD-MBA 361 cell lines, respectively (Fig. 9). One of the reasons for increasing the cytotoxic effects of niosomes containing Zn-NPs is the binding of niosomes with the cell membrane, the controlled and targeted release of the drug into the cell^41^. Rezaie Amale, et al., evaluated the cytotoxicity of AuNPs loaded into niosomes with free AuNPs on cancer cells. The researchers stated that the IC50 value in niosomes was much lower than that of nanoparticles (2.62 ± 200 µg/ml and 3.25 ± 155 µg/ml, respectively) and the results exhibited that the effects of niosomes containing AuNPs have increased^21^. Haddadian et al. compared the effects of selenium nanoparticles loaded in niosomes with free selenium nanoparticles. The results showed that when selenium nanoparticles are loaded into niosomes, their anticancer properties increase, which is due to the targeted delivery of the drug into the cell^27^.

In addition, the cytocompatibility of synthesized niosomes, niosomes containing Zn-NPs, free Zn-NPs, and free niosomes on the normal HEK-293 cell line was evaluated. As the results show, empty niosomes failed to inhibit cell growth in the HEK-293 cell line, and also when Zn-NPs are enclosed inside niosomes, the cytotoxicity is greatly reduced, which shows the biocompatibility of niosomes. it is important that when the concentration of niosomes containing Zn-NPs increases, it can have toxicity for cells, and therefore it can be concluded that lower concentrations should be used for cytotoxicity studies. Most reports show that Zn-NPs can enter the cell through membrane channels, cell membrane, or transport proteins, also through endocytosis, and can endanger the function of some organelles such as mitochondria. Most of the studies attribute the cytotoxicity of Zn-NPs to oxidative stress, lipid peroxidation, and ROS generation, which leads to DNA mutation, breakage, and finally DNA destruction^42^.

### Impact of niosomes on apoptotic genes

**Figure 10:**
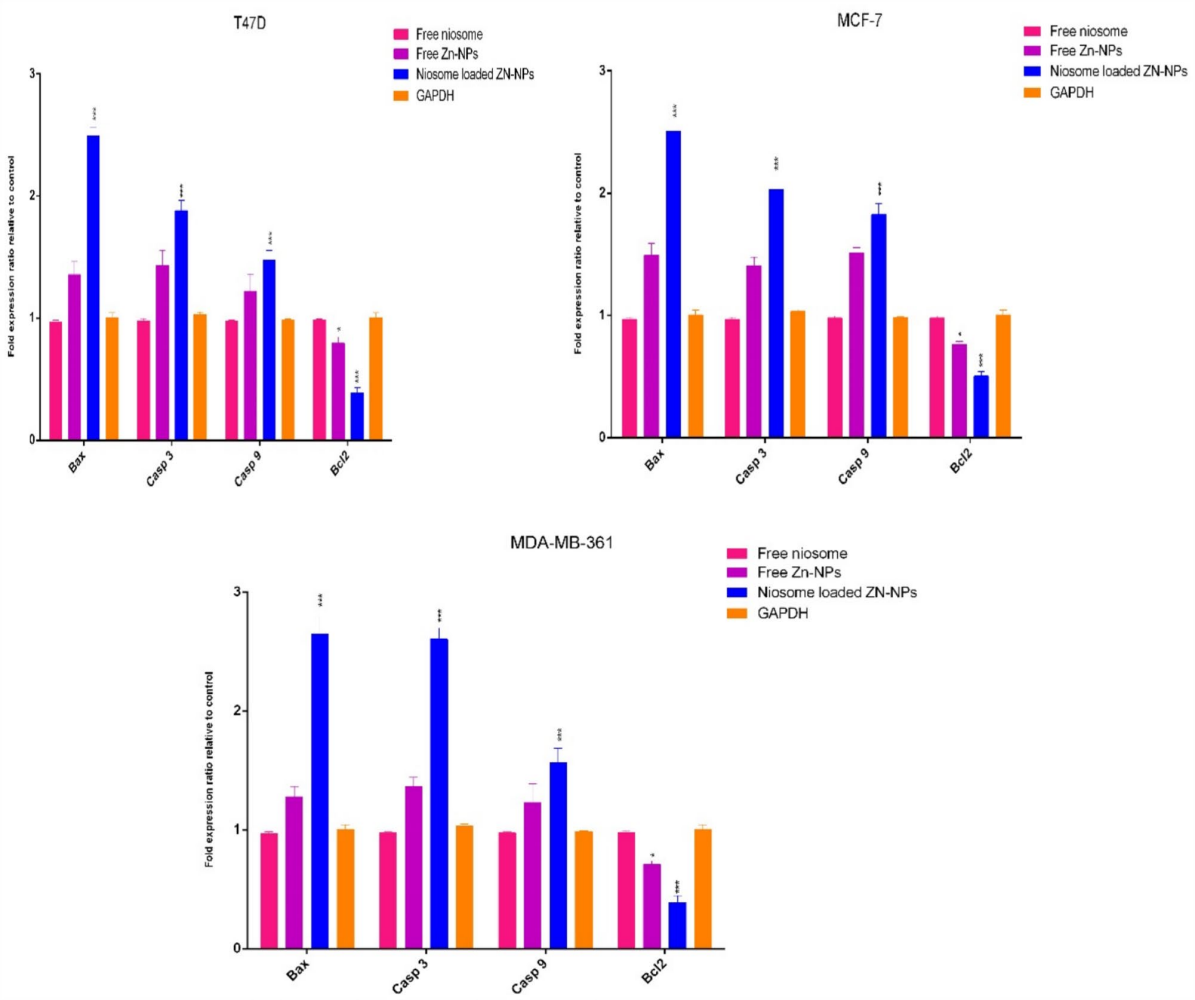
Measurement of gene expression in cells exposure to niosome-loaded Zn-NPs, free Zn-NPs, and free niosomes. As expected, the apoptotic gene expression in the cells that were exposed to niosomes increased significantly and can lead the cell to apoptosis. (*p* < 0.001***, *p* < 0.05*, n = 3)**.**

One of the methods of studying the induction of apoptosis in cells is to examine the expression of apoptotic-related genes. In the current study, 4 genes related to the process of apoptosis including *Bax*, *casp3*, *casp9*, and *Bcl2* were selected and their expression levels were investigated after treatment with niosomes containing Zn-NPs and free Zn-NPs. We observed that *Casp3* and *Casp9* apoptotic gene expression was increased in treated cells. Generally, when caspase is activated, it can activate other genes related to the caspase family, which leads to rapid apoptosis in the cell. As seen in Fig. 10, when cancerous cells are exposed to niosomes loaded Zn-NPs, the expression levels of *Bax*, *Cas3*, and* Casp9* genes up-regulated (2.41 ± 0.2, 1.87 ± 0.1 and 1.49 ± 0.3, respectively), and *Bcl2* gene expression decreases by 0.39 ± 0.05, which indicates the induction of apoptosis. The point that is worth mentioning is that many studies consider the cause of apoptosis to be the creation of ROS in treated cells, which destroys the structure of DNA and cellular components^43^.

Pouresmaeil et al., showed that Zn-NPs can induce apoptosis in cancer cells through ROS, and RT-PCR results showed that Zn-NPs can upregulate the *Bax* mRNA level (*p* < 0.001) and decrease *Bcl2* genes (*p* < 0.05). Akhtar et al., showed that Zn-NPs can increase the *Bax* and *P53* gene expression in HepG2 cells and downregulate the antiapoptotic *Bcl2* genes mRNA level, and also Zn-NPs can lead to the activation of caspase 3, activate toxic oxygen radicals and oxidative stress^44^. Cheng et al., prepared Zn-NPs by the green synthesis method and investigated their apoptotic effects on bone cancers. The results demonstrated that Zn-NPs can increase ROS generation and decrease *MMP* gene expression. Also, the expression of apoptotic genes *Bax*, *Casp3*, and *Casp9* increases, which indicates the induction of apoptosis by Zn-NPs nanoparticles^45^. Efati et al., synthesized Zn-NPs using *Lepidium sativum* L. seed extract and investigated its anticancer and apoptotic effects against human colorectal cancer cells. The researchers found that the Zn-NPs increased the expression of apoptotic genes (*P53*) and decreased the expression of *Bcl2* gene^46^. The findings are consistent with the results of our research, which shows the induction of apoptosis and the increase in the expression of apoptotic genes by Zn-NPs.

## Conclusion

In this study, using the green synthesis method, zinc oxide nanoparticles (Zn-NPs) were prepared and loaded into the niosomes. Subsequently, 6 formulations of niosomes loaded Zn-NPs were synthesized and the formulation that had a smaller size and more EE% was considered as the optimal formulation. The optimal formulation was evaluated in terms of stability and release. The synthesized niosomes were characterized by SEM, FTIR, and DLS tests and the results showed that the synthesized niosomes have suitable characteristics. Also, the optimized form of synthesized niosome showed controlled drug release. Its antimicrobial efficiency on some microbial pathogens was investigated, and its cytotoxic effects on different types of breast cancer cells were also evaluated. The results of the antimicrobial tests showed that the synthesized niosomes can increase the antimicrobial effects by 2 to 4 times. Also, the results of cytotoxicity tests showed that synthesized niosomes can have significant cytotoxicity on T47D, MCF-7, and MD-MBA 361 breast cancer cell lines including. Also, the synthesized niosomes did not have significant cytotoxic effects against normal HEK-293 cell lines which shows its biocompatibility. Overall, our findings exhibited that niosomes enhanced the biological potential of free Zn-NPs and by targeting niosomes, drugs can be directed to the target cell with a higher concentration and therefore can be a suitable carrier for targeted delivery of Zn-NPs.

## Data Availability

The datasets used and/or analyzed during the current study are available from the corresponding author on reasonable request and can be available from the corresponding author on request.
